# Outcomes of patients with acute myeloid leukemia and bone marrow fibrosis

**DOI:** 10.1186/s13045-024-01630-w

**Published:** 2024-11-15

**Authors:** Samuel Urrutia, Hagop M. Kantarjian, Farhad Ravandi-Kashani, Carlos Bueso-Ramos, Rashmi Kanagal-Shamanna, Elias Jabbour, Guillermo Montalban-Bravo, Nicholas J. Short, Naval Daver, Gautam Borthakur, Courtney D. Dinardo, Tapan M. Kadia, Lucia Masarova, Prithviraj Bose, Naveen Pemmaraju, Guillermo Garcia-Manero, Koji Sasaki

**Affiliations:** 1 Division of Oncology, Washington University in St Louis, St Louis, 200233 USA; 2 Department of Leukemia, The University of Texas MD Anderson Cancer Center, Houston, USA; 3 Department of Hematopathology, The University of Texas MD Anderson Cancer Center, Houston, USA

**Keywords:** Bone marrow fibrosis, Acute myeloid leukemia, Prognostication

## Abstract

The outcomes of patients with acute myeloid leukemia (AML) and bone marrow fibrosis (MF) are not well defined. The study objectives were to evaluate the degrees of MF in AML, and corresponding response rates and outcomes. We performed a retrospective review of 2302 patients with AML. We annotated the clinical and molecular characteristics, response to therapy, and survival outcomes of patients with bone marrow fibrosis. Overall, 492 patients (21.4%) had a reported microscopic evaluation of MF: 344 (69.9%) had MF grade 0–1 and 148 (30.1%) had MF grade 2–3. Patients with MF 2–3 had a higher proportion of complex cytogenetics (39.2% vs. 24.7%, *p* = 0.002) *JAK2* mutations (25.7% vs. 18%, *p* = 0.07) and lower proportion of *IDH2* (16.9% vs. 25.9%, *p* = 0.03) and *CEBPA* (15.5% vs. 27.6%, *p* = 0.006) mutations. 64% were treated with low-intensity chemotherapy (LIT) and 36.1% with intensive chemotherapy (IT). The complete remission (CR)/CR with incomplete count recovery (CRi) rates were 63.5% with IC versus 37.9% with LIT (*p* = 0.007). In patients aged 60 or older 4-week mortality was 12.5% with IC vs. 9.3% with LIT (*p* = 0.8). The median overall survival (OS) was 14.2 with MF 0–1 versus 7.5 months with MF 2–3 (*p* < 0.005). In patients aged 60 or older with MF 2–3 median OS was 6.5 months with IT versus 7.0 months with LIT (*p* = 0.19). In a multivariate analysis, grade 2–3 MF (HR 2.0, 95%CI 1.59–2.51) was the strongest prognostic factor for survival. In summary, grade 2–3 MF in AML is associated with worse outcomes.

**Supplementary Information:**

The online version contains supplementary material available at 10.1186/s13045-024-01630-w.

## Introduction

Acute myeloid leukemia is an aggressive hematopoietic neoplasm [1], usually treated with a combination of intensive chemotherapy and, when indicated, an allogeneic hematopoietic stem-cell transplantation (HSCT) in first remission to achieve long-term disease-free survival [2]. Multiple studies have reported the presence of cytogenetic abnormalities and molecular alterations that impact the outcomes in AML [3–5]. Currently, the risk stratification and treatment of AML relies on the presence of specific cytogenetic and molecular features which have been codified into the European Leukemia Net classification 2022 [6]. This risk schema has been recently challenged given the heterogenous outcomes of patients in multiple datasets treated with lower-intensity therapies or those in the unfavorable category [7–9]. 

Mesenchymal cells are ontogenically related to hematopoietic cells and have been postulated to arise from a common multipotent progenitor [10]. They have been found to contain similar genetic aberrations as the ones described in myeloid neoplasms [11]. The damage of mesenchymal and stromal cells in the bone marrow micro-environment leads to fibrosis and niche disruption, decreasing repopulation and tolerance to HSCT [12]. In solid tumors, the degree of tissue fibrosis or tumor stiffness has been correlated with survival [13, 14]. In myelodysplastic syndrome, bone marrow fibrosis has been associated with poor outcomes and recognized as an independent entity by the World Health Organization 2022 classification of myeloid malignancies [15]. These data suggest that the interrelationship between bone marrow fibrosis and neoplastic hematopoiesis is highly relevant in myeloid diseases but understudied in AML.

Two studies have found marrow fibrosis (MF) to be a poor prognostic factor. In AML treated with intensive chemotherapy (IT), MF was associated with a lower rate or complete response (CR) and worse survival [16]. After HSCT, MF was associated with delayed engraftment of neutrophils and platelets [17]. The role of bone marrow fibrosis in AML has not been well described and its molecular underpinnings are unknown, as are the long-term outcomes after treatment with novel therapies. The objective of this study is to describe the clinico-pathologic characteristics and outcomes of patients with AML and bone marrow fibrosis and provide features that inform their survival.

## Methods

### Study design

This study was a single-center retrospective analysis from 2007 to 2023 performed at The University of Texas MD Anderson Cancer Center (MDACC) that included patients referred with newly diagnosed AML and with annotation of bone marrow MF by a certified hematopathologist in a CLIA-certified laboratory. Reporting of bone marrow fibrosis was done for 492 patients included in the final analysis. Cytogenetic analysis was performed using conventional karyotyping and fluorescence in situ hybridization. The following cytogenetic abnormalities were grouped under MDS cytogenetics: +8, + 19, -7, -5, del7, del5, -17, and del17p. Mutations were annotated using several panels across the years ranging from 28 genes from years 2014–2017, later expanded to 81 genes from 2017 onwards. This study was conducted in accordance with the Declaration of Helsinki and approved by the MDACC review board.

### Assessment of bone marrow fibrosis

Bone marrow fibrosis was graded using the WHO system for the degree of MF [18]. Sections of formalin-fixed and paraffin-embedded bone marrow were stained with silver impregnation following Gordon-Sweets’ method. We used Masson trichrome staining and hematoxylin-eosin stains to assess for reticulin fibers, and amount of fibrosis, respectively. Based on a preliminary analysis of survival of all patients (Fig. S6), we separated patients into MF 0–1 and MF 2–3 for further analysis.

### Statistical analysis

Summary statistics were described using Pearson’s chi-squared to compare proportions and Kruskal-Wallis test for differences in medians and interquartile ranges. The median follow-up and survival times were evaluated using the Kaplan-Meier estimates. Overall survival (OS) was measured from the date of diagnosis to death. To estimate the relative effect of specific features we used the Cox proportional hazards regression in univariate and multivariate analyses with an alpha threshold of 0.05. All analyses and graphic renderings were performed using Python 3.12 (Python Software Foundation, Wilmington, Delaware, USA).

## Results

### Patient characteristics

**Fig. 1:**
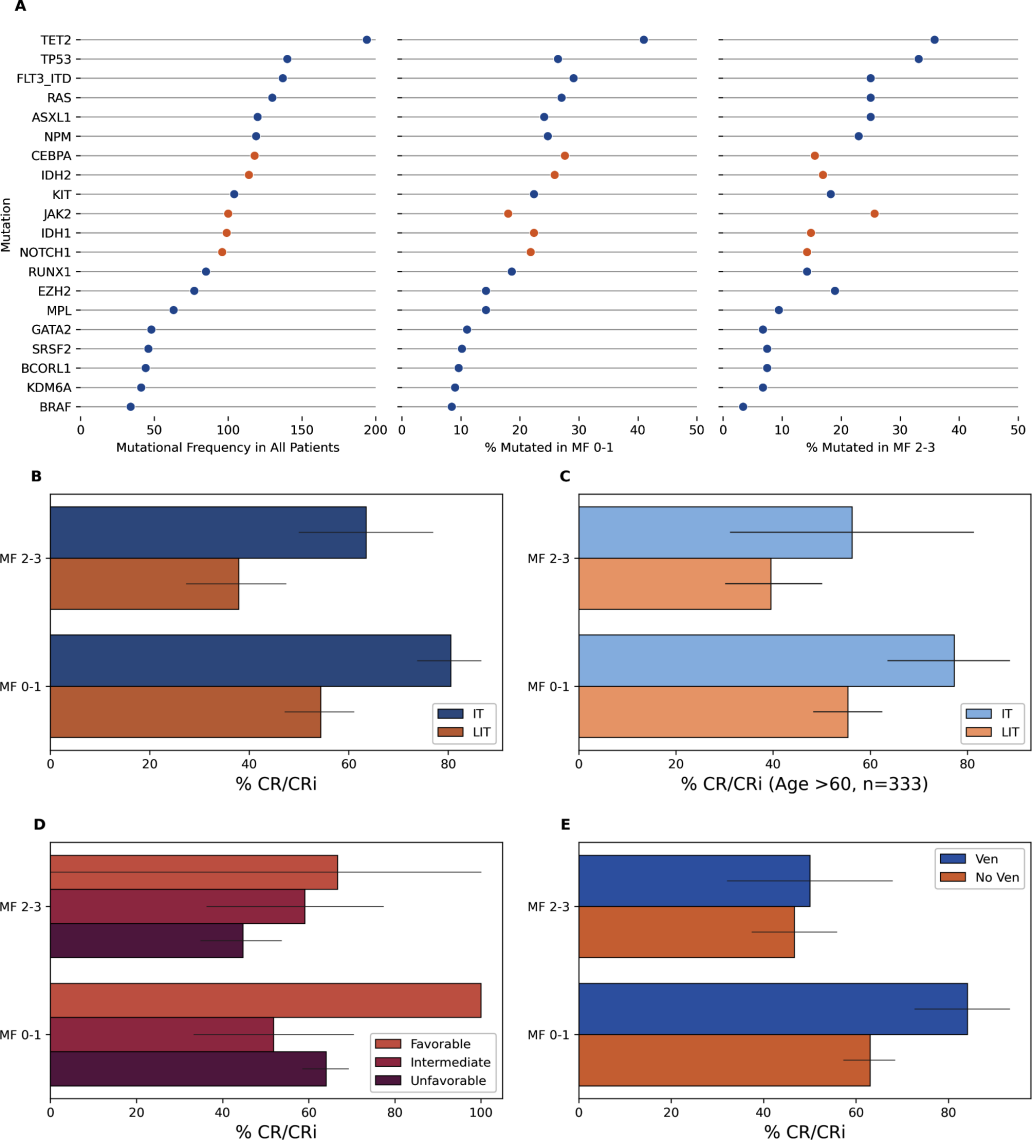
Mutational burden and response dynamics. (**A**) Left: total frequency of mutations observed in the cohort. **Center**: Mutations observed in patients with AML with MF 0–1. Right: Mutations observed in patients with AML and MF 2–3. Mutations with a statistically significant difference between the two MF groups are highlighted in orange. (**B**) Bar plots and confidence intervals of complete remission or complete remission with incomplete hematologic recovery (CR/CRi) rates by intensive therapy (IT) or low-intensity therapy (LIT). (**C**) Rates of CR/CRi in a subset of patients older than age 60 by therapy intensity. (**D**) Rates of CR/CRi in patients stratified by the ELN 2022 risk criteria (**E**) Rates of CR/CRi in patients treated with or without venetoclax

Among 2302 patients with AML from 2007 to 2023, 492 had reported MF status (21.4%). We separated patients into groups exhibiting none or mild bone marrow MF (MF 0–1) and those with moderate or severe bone marrow MF (MF 2–3) by microscopic examination as described above. A total of 344 patients (69.9%) had MF 0–1 and 148 (30.1%) had MF 2–3. The patient characteristics are shown in Table 1. Patients with MF 2–3 had a higher median absolute neutrophil count, a lower percent of marrow blasts, a lower incidence of diploid karyotype, and of *CEBPA* and *IDH2* mutations. Complex karyotype, *KMT2A* mutations, and *STAT5* mutations were more common in MF 2–3 AML (Table 1; Fig. 1A). Notably, only 6 patients (1.7%) had mutations in *DNMT3A*. A history of myeloproliferative neoplasms (MPN) was noted in 37% of MF 2–3 versus 26.2% with MF 0–1 (*p* = 0.01). A subset analysis excluding those without a history of MPN did not significantly change the relationships between baseline characteristics (Table S1). Mutations associated with MPN (*JAK2*,* CALR*, and *MPL*) were not significantly different between the two groups (Fig. S1).

**Table 1:** Baseline characteristics

Parameter	Overall	MF 0–1	MF 2–3	*p*-value	
	492	344 (69.9%)	148 (30.1%)		
Female, n (%)	224 (45.5)	162 (47.1)	62 (41.9)	0.335	
Age in yr., median [Q1,Q3]	67.0 [57.0,73.0]	67.0 [57.0,74.0]	66.0 [56.8,72.0]	0.508	
WBC x10^3/L, median [Q1,Q3]	4.0 [1.9,10.5]	3.9 [1.8,10.5]	4.5 [2.0,11.1]	0.346	
ANC, median [Q1,Q3]	0.7 [0.2,2.0]	0.6 [0.2,1.7]	1.1 [0.4,3.0]	< 0.001	
Hemoglobin g/dL, median [Q1,Q3]	9.2 [8.6,9.7]	9.3 [8.6,9.8]	9.1 [8.5,9.6]	0.07	
Platelet count 10^3/L, median [Q1,Q3]	34.0 [19.0,71.5]	37.0 [20.0,69.0]	31.0 [17.0,75.5]	0.289	
PB Blasts %, median [Q1,Q3]*	15.0 [3.0,39.0]	17.0 [3.0,43.0]	10.5 [3.0,33.2]	0.055	
BM Blast %, median [Q1,Q3]	41.0 [24.0,65.0]	47.0 [26.0,69.0]	30.0 [22.0,52.5]	< 0.001	
Diploid, n (%) †	143 (29.1)	109 (31.7)	34 (23.0)	0.065	
MDS cytogenetics, n(%)	163 (33.1)	107 (31.1)	56 (37.8)	0.177	
Complex karyotype, n (%) †	143 (29.1)	85 (24.7)	58 (39.2)	0.002	
History of MPN, n (%)	145 (29.5)	90 (26.2)	55 (37.2)	0.019	
*FLT3*-ITD, n(%)	137 (27.8)	100 (29.1)	37 (25.0)	0.416	
*TP53*, n(%)	140 (28.5)	91 (26.5)	49 (33.1)	0.164	
*NPM1*, n(%)	119 (24.2)	85 (24.7)	34(23.0)	0.766	
*CEBPA*, n(%) ‡	118 (24.0)	95 (27.6)	23 (15.5)	0.006	
*IDH1*, n(%)	99 (20.1)	77 (22.4)	22 (14.9)	0.074	
*IDH2*, n(%)	114 (23.2)	89 (25.9)	25 (16.9)	0.040	
*JAK2*, n(%)	100 (20.3)	62 (18.0)	38 (25.7)	0.070	
*KMT2A*, n(%)	9 (1.8)	1 (0.3)	8 (5.4)	< 0.001	
*NOTCH1*,n(%)	96 (19.5)	75 (21.8)	21 (14.2)	0.067	
*STAT5A*,n(%)	5 (1.0)	1 (0.3)	4 (2.7)	0.030	

WBC: white blood cell, ANC: absolute neutrophil count, PB: peripheral blood, BM: bone marrow, MPN: myeloproliferative neoplasm.*n = 477, †*n* = 466, ‡*n* = 372

Most patients were classified as ELN unfavorable (81.5%). This was mostly based on the presence of *ASXL1* mutations (24.4%), spliceosome mutations (27.6%), *RUNX1* mutations (17.3%), and abnormalities in chromosomes 7 or 5 (16.1%). All patients with core-binding factor AML (2%) had MF 0–1. Patients with AML MF 0–1 had a significantly higher proportion of ELN favorable risk (7.3% vs. 2%, *p* = 0.007), but a lower proportion of ELN intermediate risk (10.5% vs. 18.2%). The incidence of ELN unfavorable risk was similar, 82.3% with MF 0–1 and 79.7% with MF 2–3 (*p* = 0.23).

### Response to therapy

Therapy for AML was divided into intensive therapy (IT) and low-intensity therapy (LIT). Out of 492 patients, 58.9% were treated with LIT, 56.7% of AML MF 0–1 patients were treated with LIT, 64.2% of patients with AML MF 2–3 were treated with LIT. A numerically lower number of patients (35.1% vs. 43.3%) with AML MF 2–3 were treated with IT (*p* = 0.08). A total of 72 (14.6%) were treated with a regimen containing venetoclax, forty-four (12.8%) of those with AML MF 0–1, and 28 (18.9%) of those with MF 2–3 (*p* = 0.10).

Among patients with AML MF 0–1, IT resulted in 80.5% CR/CRi rates vs. 54.4% in those treated with LIT (*p* < 0.001) (Fig. 1B-C). In AML MF 0–1, venetoclax containing regimens resulted in 84.1% CR/CRi rates, vs. 63% CR/CRi rates (*p* = 0.010). Among patients with AML MF 2–3, achievement of CR/CRi was higher among those receiving IT (63.5%) vs. those receiving LIT (37.9%) (*p* = 0.007). However, in patients older than 60 (*n* = 333, Fig. 1C), IT did not result in higher CR/CRi rates. Additionally, the addition of venetoclax did not have any effect on CR/CRi rates for AML MF 2–3 (50% vs. 46.7%, *p* = 0.914) (Fig. 1E). Response rates and survival by age and degree of MF is presented in Table 2.

**Table 2:** CR/CRi rates and survival by age groups

	All ages			≥ 60 years			< 60 years			
	Overall	MF 0–1	MF 2–3	Overall	MF 0–1	MF 2–3	Overall	MF 0–1	MF 2–3	
Overall	296 (60.2)	226 (65.7)	70 (47.3)	181 (54.4)	137 (59.6)	44 (42.7)	115 (72.3)	89 (78.1)	26 (57.8)	
IT	153 (76.1)	120 (80.5)	33 (63.5)	43 (71.7)	34 (77.3)	9 (56.2)	110 (78.0)	86 (81.9)	24 (66.7)	
LIT	142 (49.0)	106 (54.4)	36 (37.9)	137 (50.4)	103 (55.4)	34 (39.5)	5 (27.8)	3 (33.3)	2 (22.2)	
p	*< 0.001*	*< 0.001*	*0.007*	*0.007*	*0.013*	*0.235*	*< 0.001*	*0.003*	*0.024*	
Ven	51 (70.8)	37 (84.1)	14 (50)	43 (72.9)	34 (85.0)	9 (47.4)	8 (61.5)	3 (75.0)	5 (55.6)	
No Ven	245 (58.3)	189 (63.0)	56 (46.7)	138 (50.4)	103 (54.2)	35 (41.7)	107 (73.3)	86 (78.2)	21 (58.3)	
p	*0.06*	*0.01*	*0.91*	*0.003*	*0.001*	*0.844*	*0.351*	*1*	*1*	
OS (mo)	11.3	14.2	7.5	9.4	11.3	6.7	20.1	36.2	10.9	

CR: complete response; CRi: complete response with incomplete hematologic recovery; MF: marrow fibrosis; IT: intensive therapy; LIT: low-intensity therapy; OS: median overall survival; Ven: venetoclax; mo: months

### Survival

**Fig. 2:**
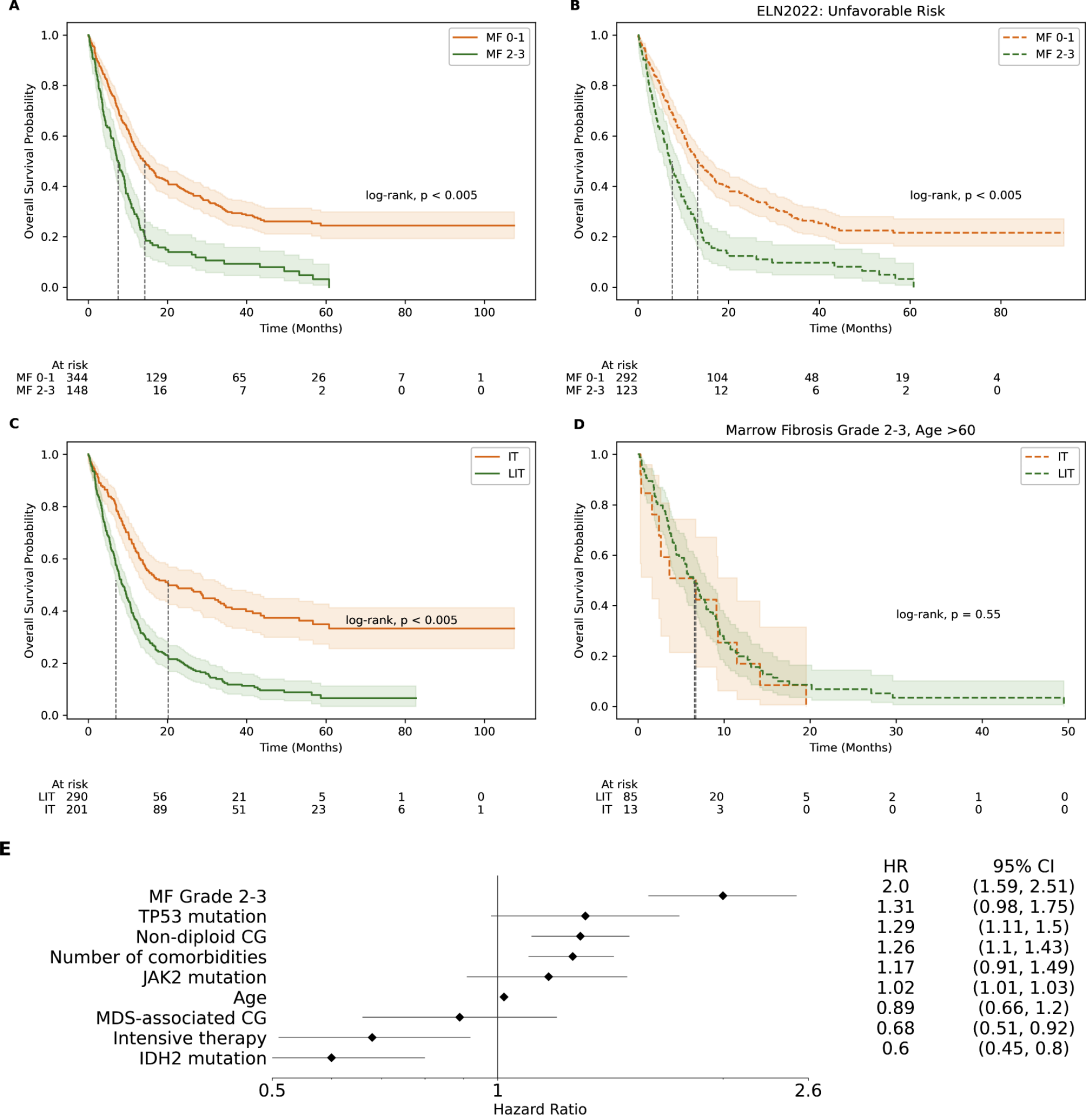
(**A**) Kaplan-Meier estimates of overall survival by degree of marrow fibrosis (MF). (**B**) Kaplan-Meier estimates of overall survival by degree of marrow fibrosis in a subset of patients classified as ELN unfavorable. (**C**) Kaplan Meier estimates of overall survival among patients treated with intensive therapy (IT) vs. low-intensity therapy (LIT). (**D**) Kaplan Meier estimates of overall survival in patients aged 60 or older who have moderate-severe (MF 2–3) bone marrow fibrosis by therapy intensity. (**E**) Hazards ratio forest plot of survival effect estimation by multivariate Cox proportional hazards model

The median follow-up time was 10.1 months (range 0.06–107 months). The median OS was 14.2 months in MF 0–1 versus 7.5 months in MF 2–3 (Fig. 2A, log-rank *p* < 0.005). The effect of MF on patients aged 60 and above was significant with a mOS of 11.3 months for patients with AML MF 0–1 vs. 6.7 months for those with AML MF 2–3 (*p* < 0.005) (Table 2). Patients with a history of MPN had a mOS of 9.4 months vs. 12.4 months for those without a prior history of MPN, but this difference did not reach statistical significance (log-rank *p* = 0.23, Fig. S2 and S4). Among patients categorized as ELN unfavorable (81.5% of total patients), those with AML MF 2–3 had a significantly lower mOS with 7 months vs. 13.2 months for those with AML MF 0–1 (Fig. 2B, log-rank *p* < 0.005), suggesting bone marrow fibrosis severity is an independent prognosticator for mortality.

Treatment intensity also affected long-term outcomes with patients receiving IT having a mOS of 33.4 months vs. 10.6 months for those receiving LIT (Fig. 2C, log-rank *p* < 0.005). For patients with AML MF 2–3, this difference was still significant but much narrower with mOS of 10.2 for those receiving IT and 6.5 months for those receiving LIT (*p* < 0.05). For patients aged 60 or older with AML MF 0–1, the survival advantage of IT is maintained with a mOS of 22.7 for IT vs. 10.6 months for the LIT strategy (*p* < 0.005). However, for patients 60 and older with AML MF 2–3, there was no difference in outcomes when exposed to IT vs. LIT with a mOS of 7 vs. 6.5 months (Fig. 2D, log-rank *p* = 0.55). The addition of venetoclax did not improve outcomes in any of the groups (Table 2, Fig S3).

### Modeling survival outcomes

In this cohort of patients with AML and bone marrow fibrosis, ELN 2022 did not properly stratify outcomes within the intermediate and unfavorable categories (Fig. 2B, S7); to resolve this, we performed Cox proportional hazard modeling to discover features correlated with survival. We found that the presence of diploid karyotype (*p* = 0.006), *IDH2* mutations (*p* < 0.001), and intensive therapy (*p* < 0.001) were correlated with longer survival, while the presence of *TP53* mutations (*p* < 0.001), complex karyotype (*p* < 0.001), MDS cytogenetics (*p* < 0.0001), older age (*p* < 0.001), and MF 2–3 ( *p* < 0.001) were correlated with worse survival. Of note, the addition of venetoclax or prior history of MPN had no effect on outcomes in this model. Using these features, we performed a multivariate analysis to better understand the interrelationships between these features. The forest plot in Fig. 2E demonstrates that MF 2–3 is the strongest predictor of survival among this subset with a hazard ratio of 2.0 (95% CI 1.59–2.51, *p* = 0.0001).

## Discussion

In this study, we evaluated patients with AML with histologic evidence of bone marrow fibrosis. We demonstrated their poor outcomes irrespective of current, clinically used risk classification criteria. These patients were treated with a combination of intensive chemotherapy or low-intensity therapy. Patients treated with intensive chemotherapy had better outcomes overall, however, in patients older than 60 years of age with evidence of grade 2–3 bone marrow fibrosis, intensive therapy did not result in higher CR/CRi rates or better overall survival. We also demonstrate that venetoclax does not improve CR/CRi rates or survival in patients with grade 2–3 fibrosis regardless of age group.

Bone marrow fibrosis has been associated with worse outcomes. In an analysis by Wu et al., density of bone marrow reticulin fiber was associated with shorter relapse-free and overall survival in 112 patients with AML in a single center in China [19]. A different study by Zhang et al. found a lower rate of CR/CRi and overall survival in 60 patients with bone marrow fibrosis treated with intensive chemotherapy [16]. In our study, *CEBPA*, *IDH1*, and *IDH2* mutations were less common in grade 2–3 fibrosis. *DNMT3A* mutations were unusually rare (6 patients), this suggests that patients with AML and MF, may either have a distinct foundational mutational event, or a clone with higher fitness (such as *JAK2*) outcompetes pre-existing *DNMT3A* clones. Despite their overall favorable prognosis, in our study, patients with AML and *NPM1* mutations and MF 2–3 had a median OS of 12 months compared to patients with 20 months for those with MF 0–1 (Fig. S5).

In patients older than 60 years with grade 2–3 fibrosis, intensive chemotherapy did not result in improved CR/CRi rates. Additionally, the use of venetoclax (*n* = 72) did not lead to higher response rates. In this study, 81% of patients were classified as ELN unfavorable risk based on the presence of *ASXL1* or spliceosome mutations. While this clearly reflects a high-risk cohort, we observed many patients had better outcomes despite their ELN classification. To explore this, we performed a univariate analysis from which features were selected for a multivariate analysis in which grade 2–3 fibrosis, abnormal cytogenetics, and number of comorbidities predicted outcomes. Overall, the use of intensive therapy also improved response and survival in the whole cohort but not in patients aged 60 or older with grade 2–3 fibrosis.

Bone marrow fibrosis has also been associated with worse outcomes and therapy failure in chronic myeloid leukemia [20], it has been proposed as a risk factor for post transplantation outcomes [21], and it defines a unique clinical and histologic entity in myelodysplastic syndromes which is enshrined in recent societal classifications [15, 22–24]. In AML, there has been no formal definition or characterization of AML with fibrosis, and up to this point this feature is not included in modern morphologic or risk classifications. Conversely, multiple studies have studied the outcomes of post-MPN or blast-phase MPN, a highly lethal condition [25]. In our study, patients with grade 2–3 bone marrow fibrosis and *de novo* AML had a survival of 12 months compared to 9 months for those with post-MPN AML, a difference that was not statistically significant. This finding suggests a unique entity with potential shared biological origin translating into mesenchymal stem cell dysfunction and potentially similar therapeutic vulnerabilities.

Current classification criteria for AML do not consider bone marrow fibrosis. Current risk strategies such as ELN do not consider the prognostic weight of bone marrow fibrosis. In our study, we demonstrate that advanced bone marrow fibrosis dictates response and survival outcomes in *de novo* AML providing evidence for consideration of AML with bone marrow fibrosis as a unique and unfavorable clinical entity.

## Electronic supplementary material

Below is the link to the electronic supplementary material.

Supplementary Material 1

Supplementary Material 2

## Data Availability

No datasets were generated or analysed during the current study.
