# Supplementary material for: Outcomes of patients with acute myeloid leukemia and bone marrow fibrosis
Source: J Hematol Oncol. 2024 Nov 15;17:112. doi: 10.1186/s13045-024-01630-w (PMC11568598; doi:10.1186/s13045-024-01630-w)

**SUPPLEMENTARY FIGURES**

Table of Contents

[Figure S1. Extensive mutational annotation by de novo vs post-MPN AML. 2](#_Toc181016794)

[Figure S2. Survival of patients with AML with grade 2-3 marrow fibrosis de novo vs post-MPN AML. 3](#_Toc181016795)

[Figure S3. Overall survival by exposure to Venetoclax. 4](#_Toc181016796)

[Figure S4. Outcomes by therapy intensity for both de novo AML and post-MPN AML. 5](#_Toc181016797)

[Figure S5. Outcomes in patients with NPM1 mutations and degree of bone marrow fibrosis. 6](#_Toc181016798)

[Figure S6. OS of all patients (n=2302) by degree of bone marrow fibrosis including patients who had no fibrosis assessment. 7](#_Toc181016799)

[Figure S7. Stratification by ELN 2022 for all patients (n = 492). 8](#_Toc181016800)

# Figure S1. Extensive mutational annotation by de novo vs post-MPN AML.


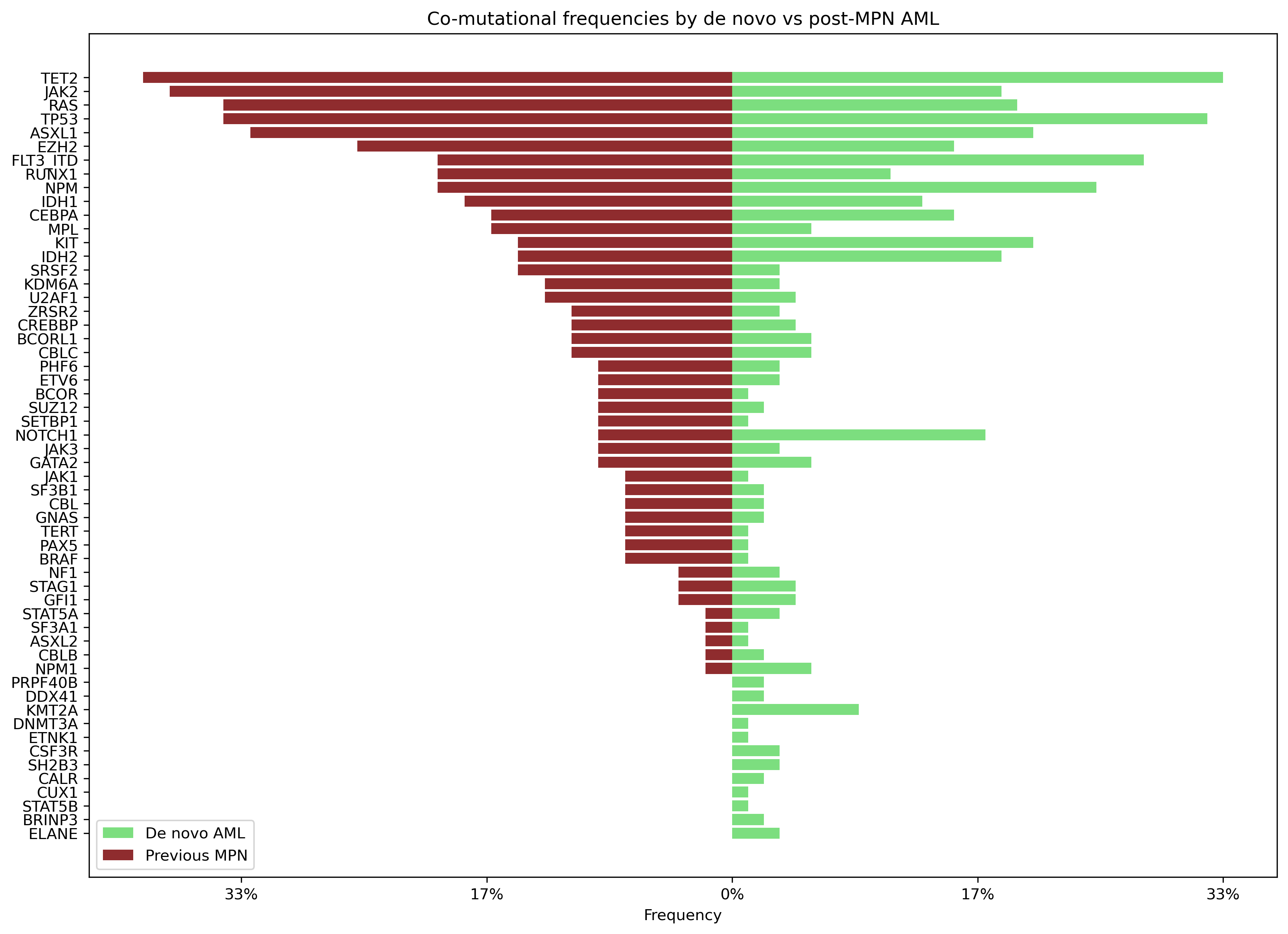


# Figure S2. Survival of patients with AML with grade 2-3 marrow fibrosis de novo vs post-MPN AML.

**
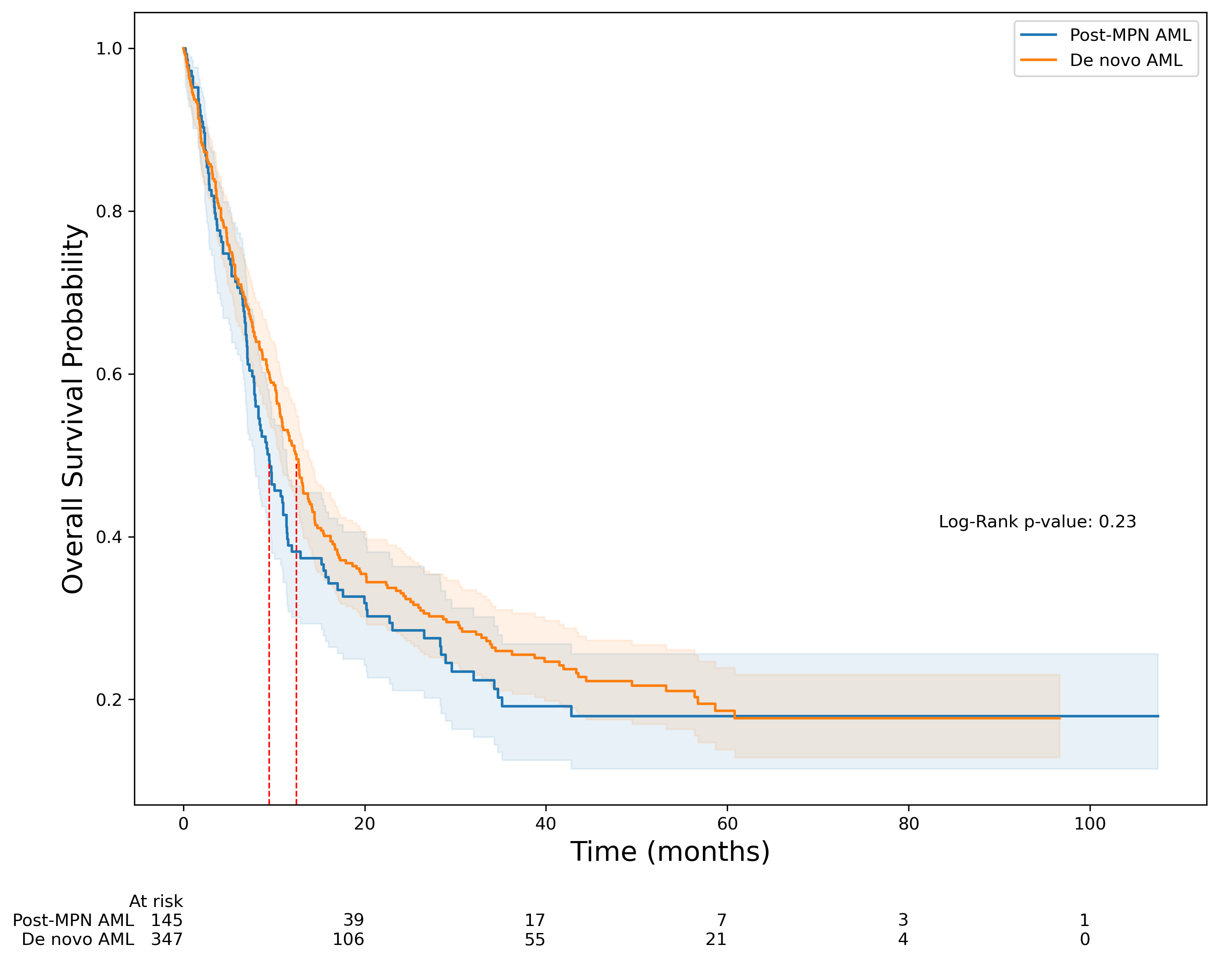
**

# Figure S3. Overall survival by exposure to Venetoclax.


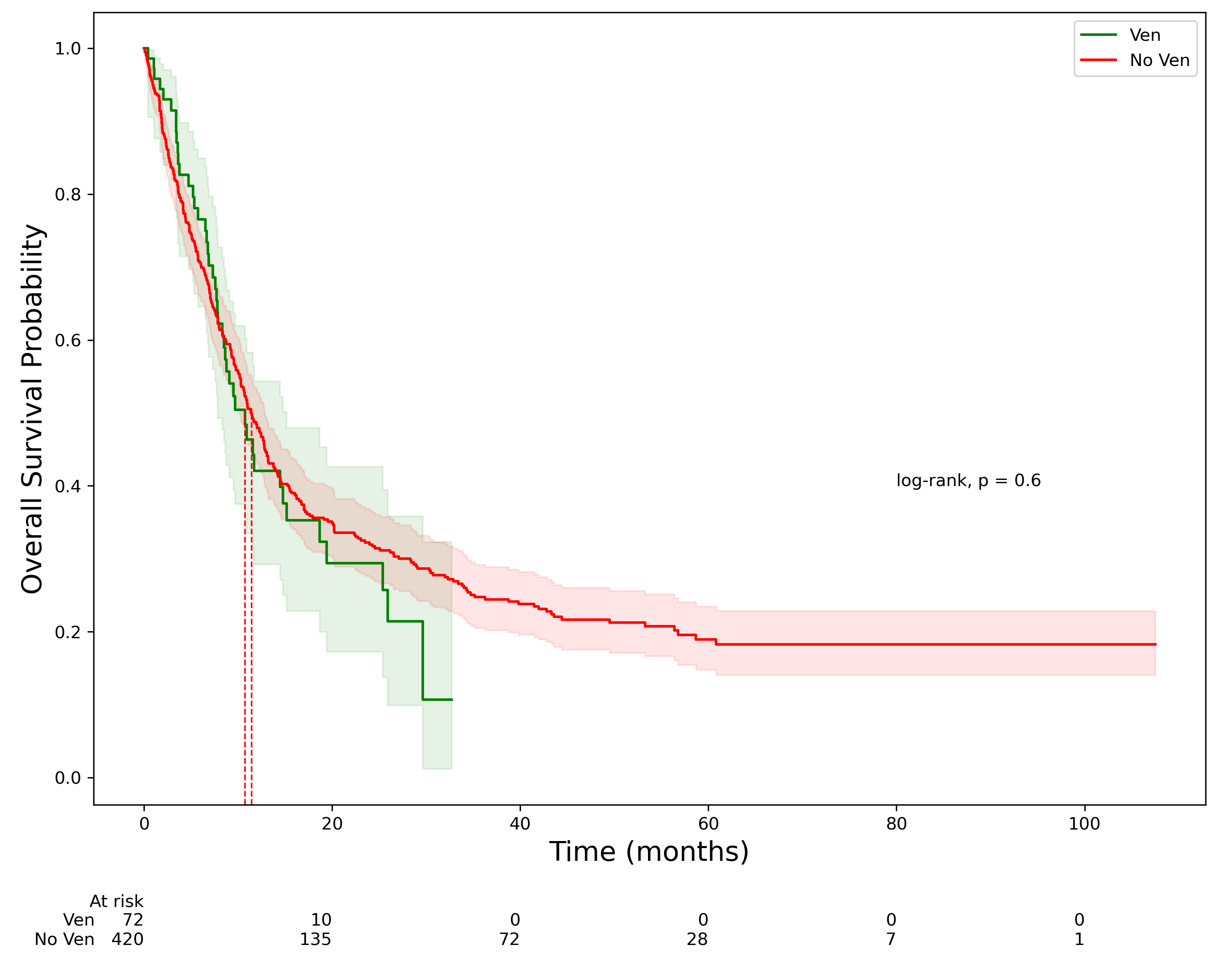


# Figure S4. Outcomes by therapy intensity for both de novo AML and post-MPN AML.


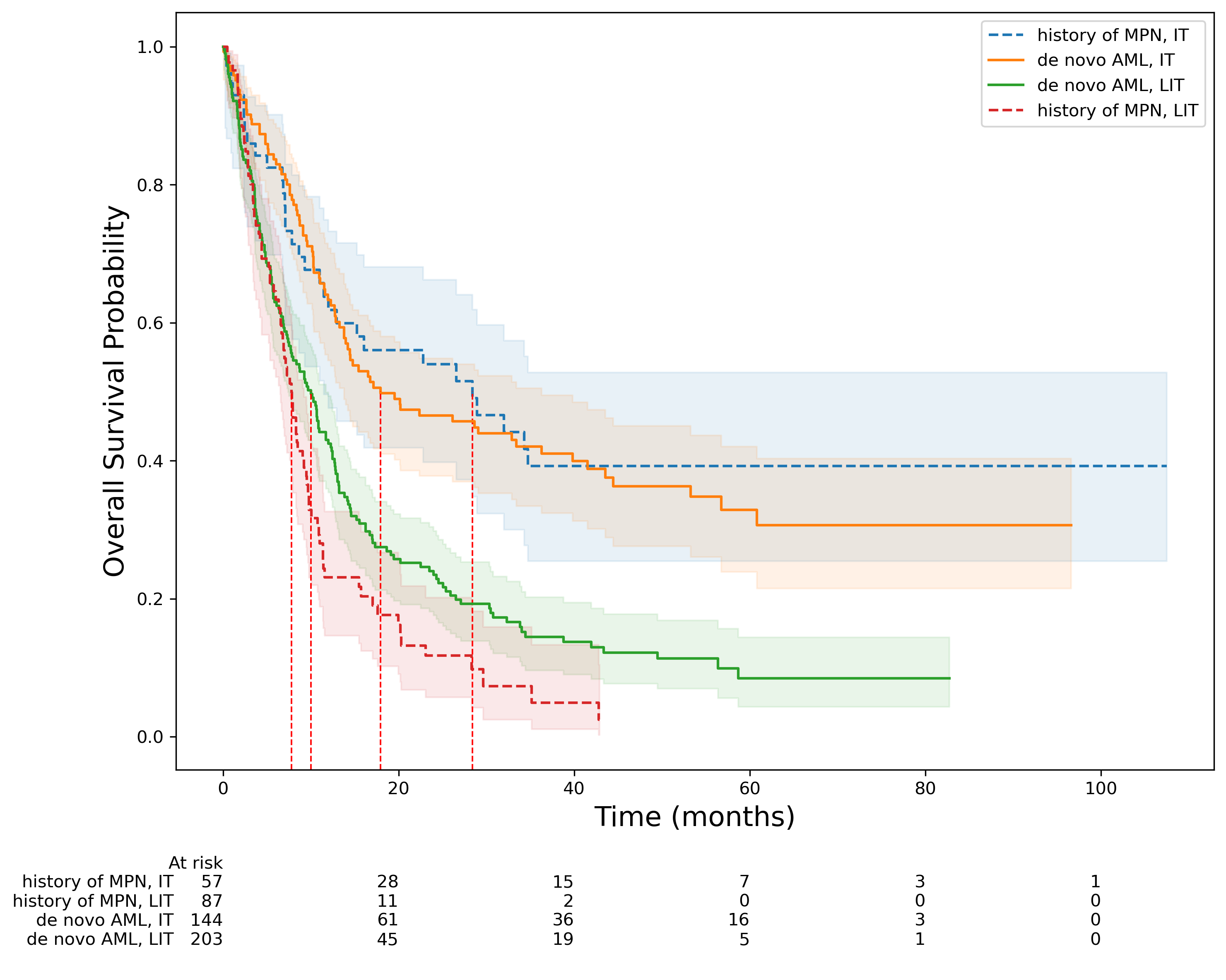


# Figure S5. Outcomes in patients with NPM1 mutations and degree of bone marrow fibrosis.


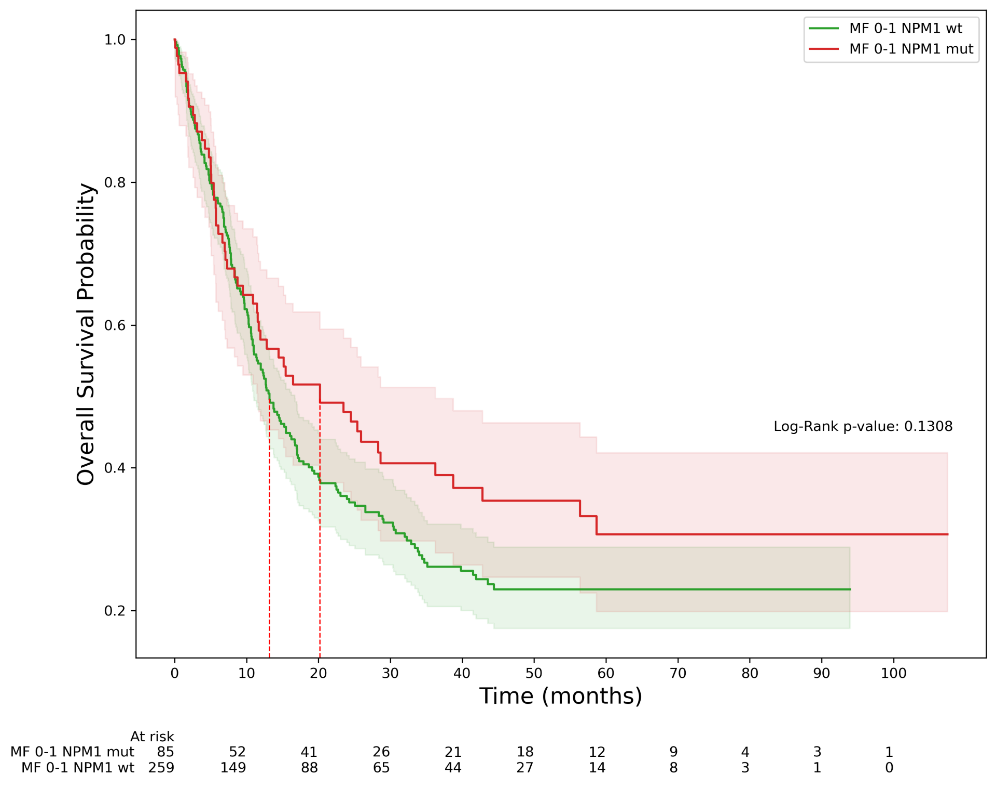


# Figure S6. OS of all patients (n=2302) by degree of bone marrow fibrosis including patients who had no fibrosis assessment.


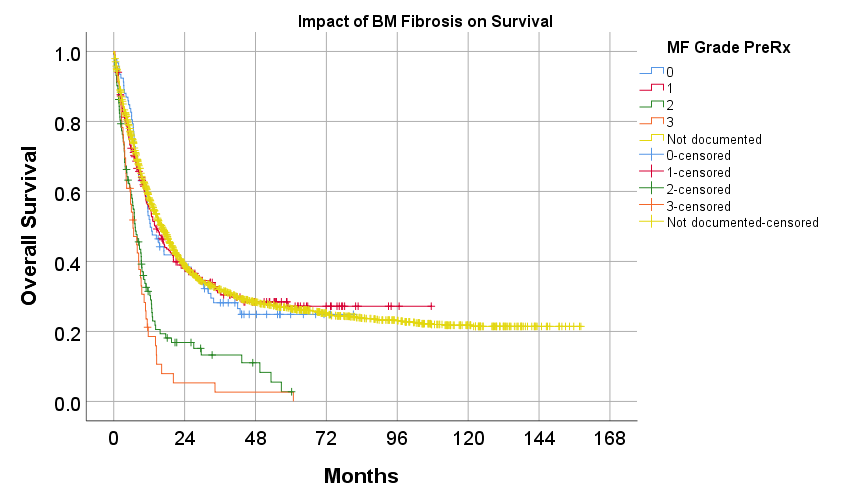


# Figure S7. Stratification by ELN 2022 for all patients (n = 492).


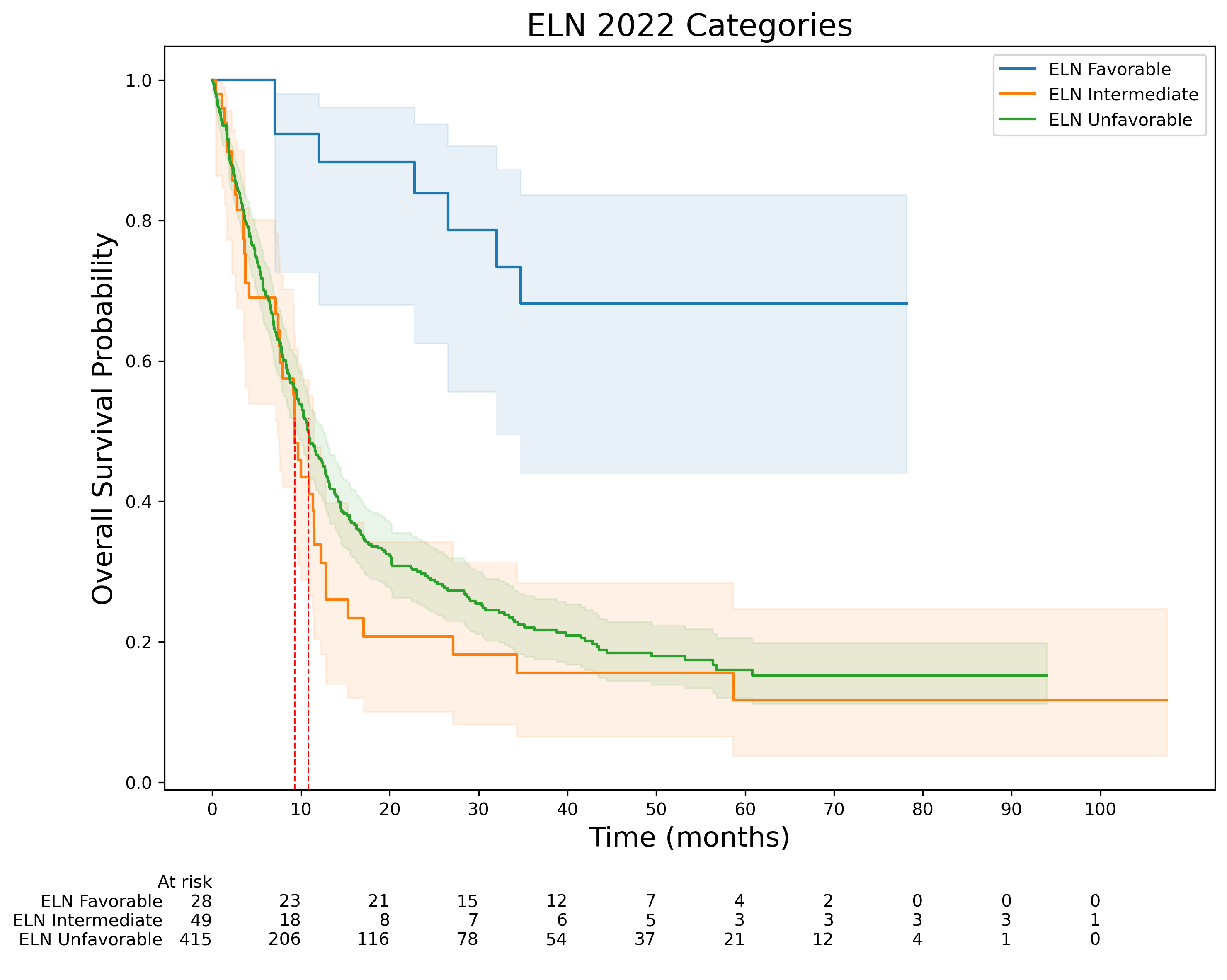

Supplement: Supplementary file 1 — Supplementary Material 1 [file 13045_2024_1630_MOESM1_ESM.docx]
